# Effects of different afforestation years on soil moisture and nutrient content in Maxian Mountain of the Loess Plateau

**DOI:** 10.1038/s41598-024-66408-z

**Published:** 2024-07-13

**Authors:** Liang Mao, Yugu Miao, Yiru Ge, Shaochong Wei, Xuanyi Yang, Shijie Li, Li Si, Yu-Ping Gou, Peter Quandahor

**Affiliations:** 1 Gansu Agricultural University, Lanzhou, 730070 People’s Republic of China; 2 Forestry and Grassland Bureau of Lintao County, Dingxi, 730075 Gansu Province People’s Republic of China; 3 CSIR-Savanna Agricultural Research Institute, P. O Box 52, Tamale, Ghana

**Keywords:** Loess Plateau, Afforestation, Age soil moisture, Soil nutrient, Grassland ecology, Restoration ecology

## Abstract

In the area of “returning farmland to forest” on the Loess Plateau in China, it is difficult to cultivate artificially planted trees into forests. In the current study, abandoned cultivated land after 10 years of natural restoration served as controls (CK), while the treatments included afforestation periods of 2, 4, 6, 8, and 10 years. Soil samples were collected from various depths: 0–20, 20–40, 40–60, 60–80, to 80–100 cm. The findings revealed that with increasing years of artificial afforestation, soil pH gradually increased, and soil moisture content rose in the 0–20 cm layer while declining in deeper layers (20–100 cm) in the Maxian Mountain region of the Loess Plateau. Moreover, the total carbon, nitrogen, phosphorus, and potassium content initially increased and then decreased with the duration of artificial afforestation, reaching peak values after 8 years. Contents of organic matter, ammonium nitrogen, nitrate nitrogen, available phosphorus, and available potassium in the same soil layer increased with each year of afforestation. However, upon reaching 10 years of artificial afforestation, the effective nutrient content in the 60–80 and 80–100 cm soil layers exhibited a decrease. The values of Integrated Fertility Index (IFI) in different afforestation years were ranked as follows: 8 years > 6 years > 10 years > 4 years > 2 year, but all of them were significantly smaller than those of natural restoration plot CK (*P* < 0.05). Overall, soil fertility in the Maxian Mountain area of the Loess Plateau increases with each additional year of artificial afforestation. However, when the artificial afforestation period is 10 years, soil fertility decreases and marking a shift from enhancement to decline beyond this duration.

## Introduction

The Loess Plateau is the world’s largest Loess accumulation area, spans 1000 km from east to west and 750 km wide from north to south, covering an area of 640,000 km^2^ with soil depths ranging from 50 to 80 m, and in some areas exceeding 250 m^1^. Positioned in the middle of China and the upper Yellow River Basin, it is recognized as one of the cradles of four major world civilizations, transitioning from nomadic to agricultural culture and nurturing the longstanding Chinese civilization. However, with the advancement of human civilization, population growth, and the conversion of native grasslands and forests to farmland^2^, coupled with persistent arid climates, excessive grazing, and long-term intensive agricultural practices^3,4^, the Loess Plateau has spiraled into a cycle of economic and ecological impoverishment, marked by getting poorer, getting more cultivated, and getting poorer^5^. Statistics reveal that in the 1980s, the Loess Plateau harbored 23 million impoverished individuals^6^, with an annual soil erosion rate of 2.233 billion tons^7^ and an annual soil organic matter loss of 18 million tons^8^. Additionally, approximately 40 million tons of nitrogen, phosphorus, and potassium^9^ were depleted from the soil, resulting in poor soil quality and low agricultural productivity^10^. Consequently, the Loess Plateau has transformed from a cradle of human civilization into an economically underdeveloped region, hindering long-term societal progress^11^.

Planted forests refer to artificial ecosystems established by planting native or exotic tree species^12^, with functions such as soil erosion prevention, ecological and environment improvement, and biodiversity enhancement^13^. In 1999, the Chinese government initiated a large-scale “returning farmland to forests” project, converting 4.83 million km^2^ of farmland into forests^14,15^. This initiative led to an increase in vegetation cover from 30% in 1982 to 45% in 2013^16^ and a significant reduction in the annual sediment input into the Yellow River, from 1.6 billion tons in the 1960s to 200 million tons presently^17^. However, due to cost and survival rate limitations, artificial forests predominantly adopt a simple, time-saving, labor-saving, and cost-effective pure forest afforestation model^18^. Spruce, *Robinia pseudoacacia* and apricots^19^ are commonly used in the Loess Plateau of China, among which spruce is widely used in afforestation projects due to its cold-resistant and evergreen characteristics. Nevertheless, as the duration of artificial afforestation increases, pure spruce forests exhibit slow growth, short stature, and weakness^20^, resulting in low ecological restoration function and canopy closure^21^, biodiversity loss, soil fertility degradation, and reduced ecosystem stability^22,23^. Therefore, effectively harnessing the ecological service functions of the Loess Plateau Grain for Green Project, such as water storage, soil moisture retention, wind and sand prevention, and soil erosion control, emerges as a pressing issue in the management and protection of artificial forests.

Soil moisture plays a critical role in both plant nutrition and the survival of soil organisms. Furthermore, soil water levels influence various physicochemical, biochemical, and microbiological processes. Babur et al.^24^ suggest that soil moisture and temperature are the primary factors significantly impacting soil biochemical properties and microbial respiration across numerous comparisons. This finding is consistent with several previous studies, which have demonstrated an increase in microbial populations under optimal soil temperature and moisture conditions^24,25^. These findings underscore the significance of soil moisture content in facilitating microbial biomass growth in temperate forests^26,27^.

The forestry ecological restoration commenced relatively late but advanced rapidly in China. Since the 1970s, up to the ninth national forest resource inventory conducted in 2019, the national forest plantation area had expanded to 80 million km^2^, ranking first globally in terms of artificial forest area, and constituting 27% of total global artificial forest area^28^. In 2020, China proposed a national strategy for ecological protection and high-quality development in the Yellow River Basin to further strengthen the important ecological barrier, incorporating the consolidation and enhancement of the Loess Plateau Grain for Green Project in the planning outline^29^. The Loess Plateau returning farmland to forest project, our research focused on afforestation mode, ecological value, carbon fixation capacity, etc., ignoring the problem that planting trees can not grow into the forest. This research was based on the hypothesis that within 10 years of afforestation, there is a turning point period of soil water and fertilizer supply capacity from positive to negative. The study was therefore conducted to identify the inflection point year of soil water and fertilizer shortage after afforestation through estimating changes of soil water and fertilizer in different afforestation years, so as to provide practical guidance for scientific afforestation.

## Research method

### Study areas

The research was conducted at the Maxian Mountain in the Loess Plateau, about 40 km south of Lanzhou City, with geographical coordinates of 35° 45′ N, 103° 58′ E, and an altitude of 2750–2860 m. The climate was cold and semi-humid, with an average temperature of − 1.4 °C, a frost-free period of 67–90 days, and an average annual rainfall of 494 mm^30^. The tested soil site was semi-dry soft soil according to US Soil Taxonomy^31^. *Rhododendron qinghaiense* and *Hippophae rhamnoides* are the dominant species, with *Lonicera hispida* and red *Arctous ruber* companions^20^.

### Sample plot settings

**Figure 1:**
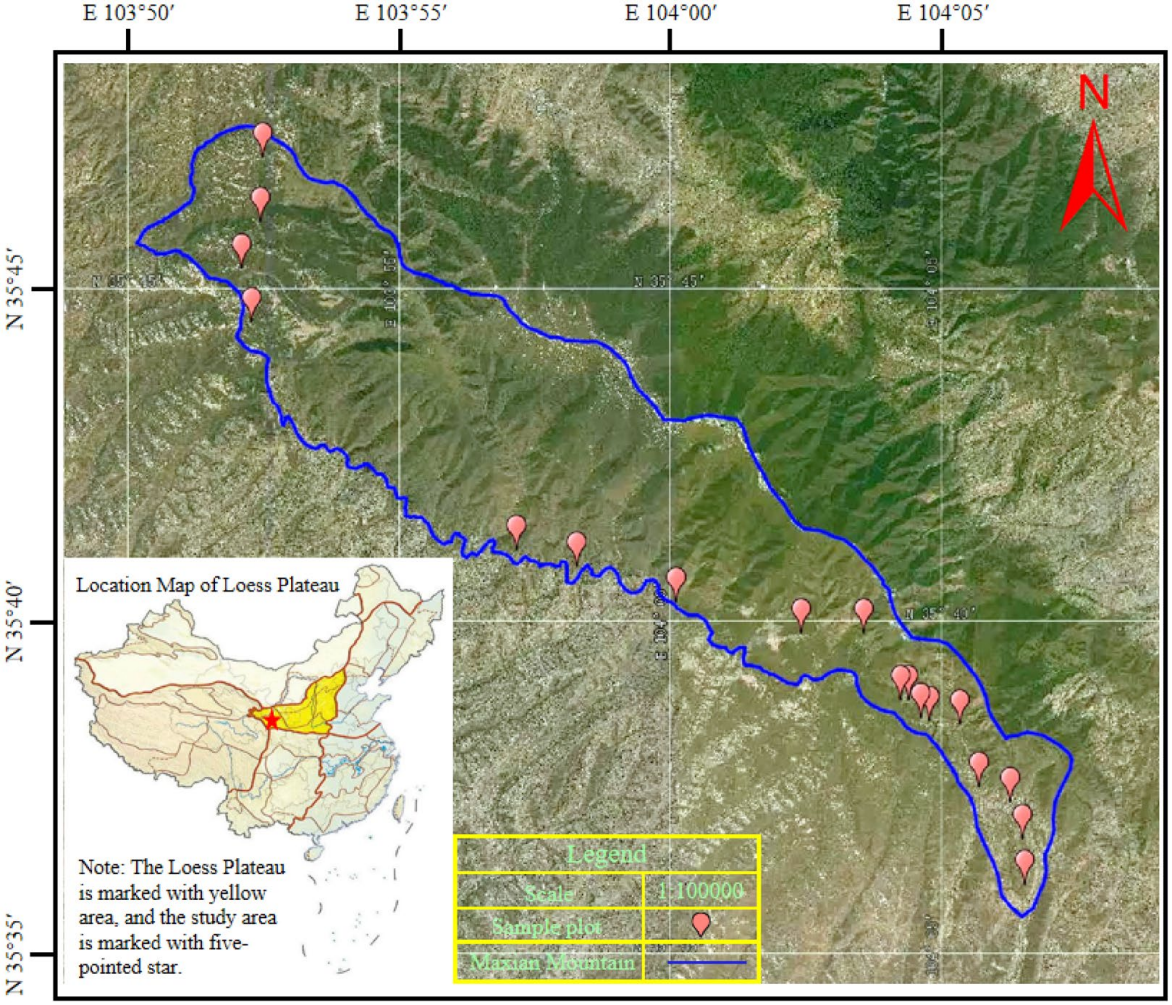
Map of research area and sample plot distribution. The location map of the Loess Plateau was from http://t.newdu.com/m/view.php?aid=2544, the satellite map was from Google Maps. Arc GIS 10.3 software was used to generate the map (URL link is https://www.esri.com/en-us/home).

The sample plot is situated on the Puyin Rural Road in Madian Mountain, Lintao County, Dingxi City, Gansu Province (Fig. 1). Afforestation projects were conducted during 2012, 2014, 2016, 2018, and 2020, saplings of *Picea crassifolia* were planted with a row spacing of 2 × 3 m and an average height of 1.5–2.0 m. The research site was fenced with iron wire. Gradients of afforestation years were established for 10, 8, 6, 4, and 2 years in succession, and then CK was installed on a sample plot of abandoned farmland that had undergone natural restoration for 10 years. The experiment involved 6 soils of afforestation year, 5 depths of soil layer, and 3 replications. A total of 6 × 5 × 3 = 90 soil samples were collected, and field sampling was completed in September 2022 (Table 1).

**Table 1:** Basic situation of sample plots with different afforestation years in Maxian Mountain of the Loess Plateau.

Plot No	Afforestation (year)	Geographic coordinates	Aspect	Altitude (m)
1	CK	35° 38′ 48″ N 104° 04′ 5″ E	North	2790
2	CK	35° 38′ 26″ N 104° 05′ 11″ E	Northwest	2719
3	CK	35°46′ 01″ N 103° 52′ 19″ E	North	2632
4	10	35° 40′ 17″ N 103° 59′ 58″ E	North	2775
5	10	35° 37′ 16″ N 104° 06′ 06″ E	Northwest	2628
6	10	35° 38′ 48″ N 104° 04′ 14″ E	Northwest	2776
7	8	35° 44′ 31″ N 103° 52′ 09″ E	Northeast	2738
8	8	35° 36′ 43″ N 104° 06′ 20″ E	North	2584
9	8	35° 38′ 32″ N 104° 04′ 28″ E	Northeast	2766
10	6	35° 36′ 02″ N 104° 06′ 22″ E	Northwest	2606
11	6	35° 39′ 49″ N 104° 03′ 25″ E	Northwest	2846
12	6	35° 45′ 19″ N 103° 51′ 58″ E	North	2704
13	4	35° 46′ 59″ N 103° 52′ 21″ E	Northwest	2804
14	4	35° 38′ 29″ N 104° 04′ 37″ E	North	2765
15	4	35° 40′ 50″ N 103° 58′ 08″ E	Northeast	2710
16	2	35° 37′ 30″ N 104° 05′ 32″ E	North	2595
17	2	35° 39′ 49″ N 104° 02′ 16″ E	Northeast	2783
18	2	35° 41′ 05″ N 103° 57′ 01″ E	Northwest	2694

### Sample analysis

Soil samples in an aluminium box were dried in a laboratory oven at 105 °C, weighed, and measured to determine moisture content. The soil sample inside the self-sealing bag was brought indoors for air drying, sieved, and converted into a test soil sample for experimental analysis. The soil pH was determined using the potentiometric method with PHSJ-6L. The total carbon and nitrogen levels in the soil were determined using the dry burning method and the Flash EA 1112 elemental analyzer.The organic matter was determined using the potassium dichromate external heating method. KCI was used to extract nitrate nitrogen and ammonium nitrogen, which were then measured with an FIA star 5000 flow injection analyzer^32^. Total phosphorus and available phosphorus were boiled with citric acid and extracted with NaHCO_3_, respectively. The molybdenum antimony colorimetric method was used, and the UV-2355 spectrophotometer was used for the assessments. Total potassium and available potassium were melted with NaOH and extracted with NH_4_OAc, respectively; Model 2655–00 flame photometer was used as reported by Zhang et al^33^.

### Metric calculations

The Integrated Fertility Index (IFI) is the synthesis and integration of various fertility factors in soil^34^.

$$IFI = \sum\limits_{i = 1}^{n} {W_{i} \times F(X_{i} )}$$

where *W*_*i*_ represents the weight of each fertility factor, *F*(*X*_*i*_) means membership value of each fertility factor. $W_{i} = Capacity/\sum\nolimits_{i = 1}^{n} {Capacity}$, where in $Capacity_{i}$ the principal component factor load of the *i* soil fertility indicator.

1 $$F(x_{i} ) = (x_{ij} - x_{i\min } )/(x_{i\max } - x_{i\min } )$$

2 $$F(x_{i} ) = (x_{i\max } - x_{ij} )/(x_{i\max } - x_{i\min } )$$

The formula, *x*_*ij*_ represents the average value of various fertility indicators, while *x*_*i*max_ and *x*_*i*min_ represent the maximum and minimum values of fertility indicators, respectively. Based on the positive and negative loading of the principal component factors, the ascending and descending properties of the membership function were determined. Nutrient indicators such as soil moisture content, carbon, nitrogen, phosphorus, and potassium were calculated using the ascending function Eq. 1, while soil pH is calculated using the descending function Eq. 2.

### Statistical analysis

Data were analyzed by the analysis of variance using SPSS statistics software (Version 19.0 for Windows, SPSS, Chicago, IL, USA). Treatment means were separated using Duncan’s multiple range test (*P* < 0.05). The results are presented as mean ± SD.

## Result

### Effect of afforestation years on soil moisture content

**Figure 2:**
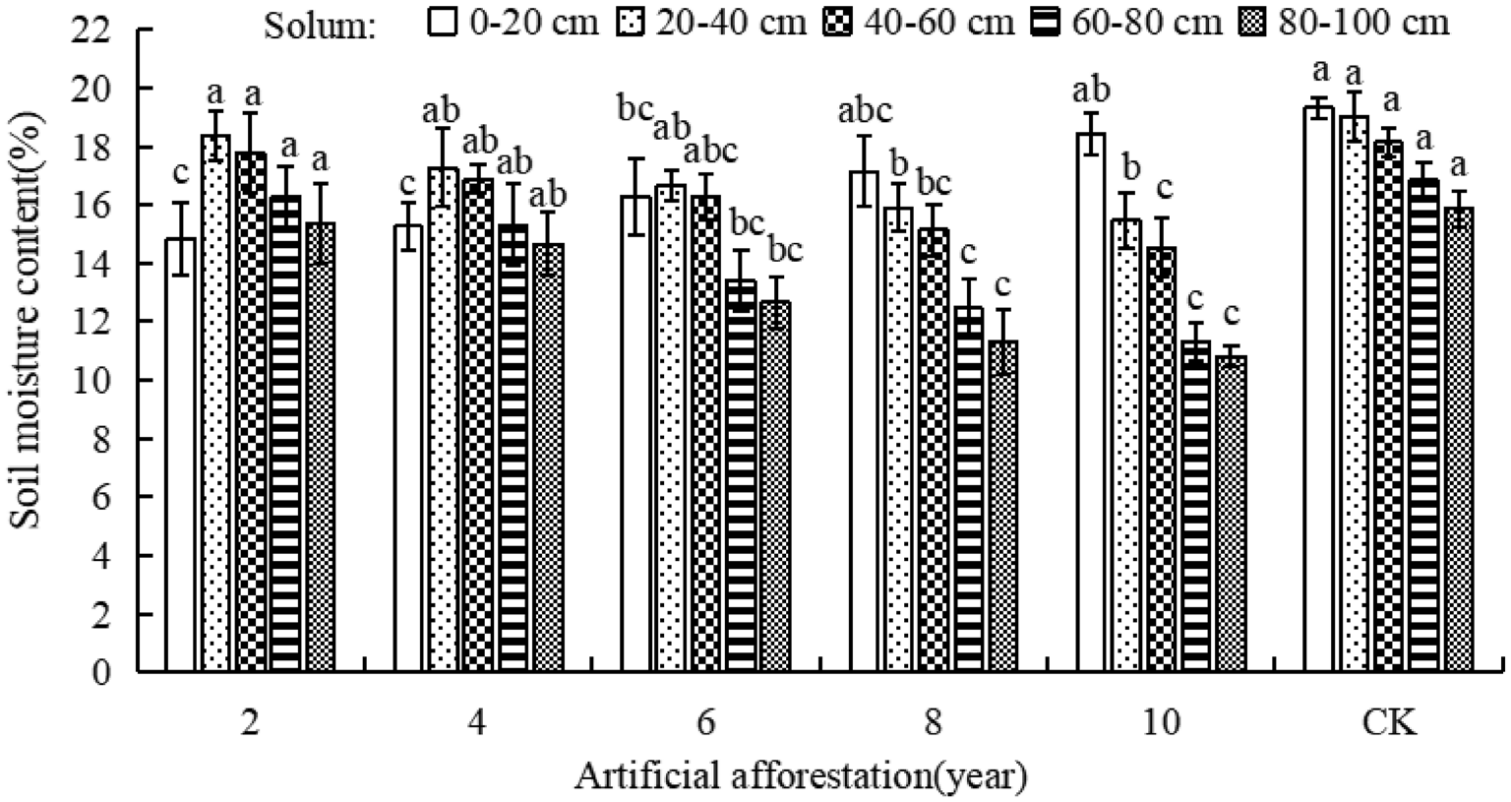
Soil moisture content of different afforestation years in Maxian Mountain of the Loess Plateau. Different lowercase letters indicate significant differences in soil moisture content at the *P* < 0.05 level between different years of afforestation in the same soil layer.

As the afforestation years increases, the soil moisture content in the 0–20 cm soil layer in the Maxian Mountain of the Loess Plateau gradually increased, while the soil moisture content in the 20–40, 40–60, 60–80, and 80–100 cm soil layers gradually decreased (Fig. 2). When the afforestation period was 2, 4, and 6 years, the soil moisture content of the 0–20 cm soil layer was significantly lower than that of CK. Afforestation of 8 and 10 years resulted in a significant reduction in soil moisture content in soil layers of 20–40, 40–60, 60–80, and 80–100 cm compared with CK (*P* < 0.05). This indicates that with the increase of afforestation years, the soil moisture content 0–20 cm soil layer increased and approached CK, while it continued to decrease in the soil layers of 20–40, 40–60, 60–80, and 80–100 cm, and the soil moisture content decreased with increase of soil depth.

### Effect of afforestation years on soil pH

**Figure 3:**
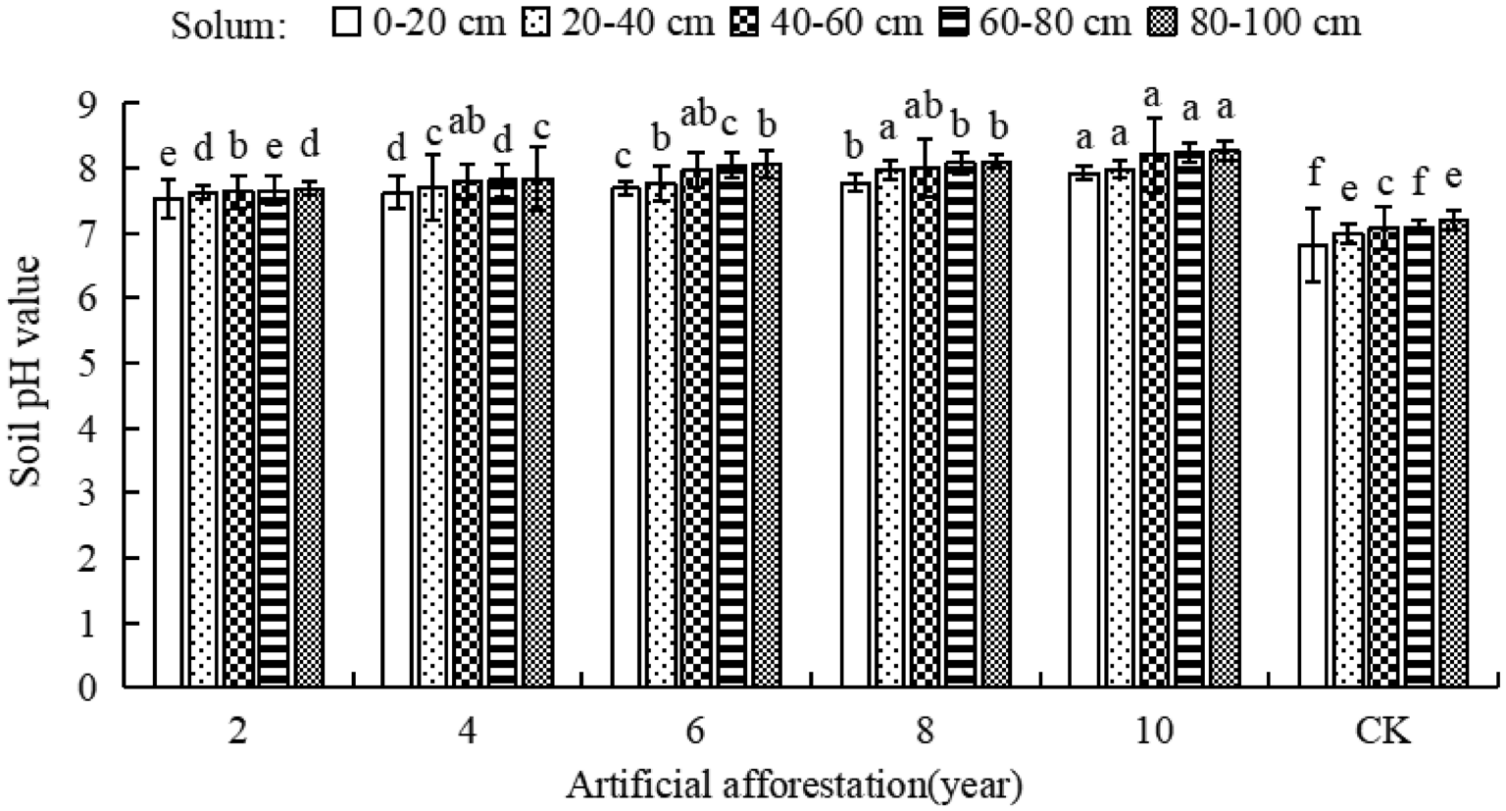
Soil pH of different afforestation years in Maxian Mountain of the Loess Plateau. Different lowercase letters indicate significant differences in soil pH at the *P* < 0.05 level for the same soil layer but different years of afforestation.

In Maxian Mountain of the Loess Plateau, with the increase of afforestation years, the soil pH of the same soil layer increased. The soil pH was highest when the afforestation years were 10 years (Fig. 3). The soil pH was significantly higher than CK (*P* < 0.05) in soil layers of 0–20, 20–40, 40–60, 60–80, and 80–100 cm with different years of afforestation. When the afforestation period is 2, 4, 6, 8, and 10 years, the soil pH increased, which was consistent with the CK variation pattern. This indicates that as the number of afforestation years increases, so does the soil pH of the same soil layer, but it does not change the vertical variation pattern of soil pH with the same afforestation years.

### The impact of afforestation years on soil carbon

**Figure 4:**
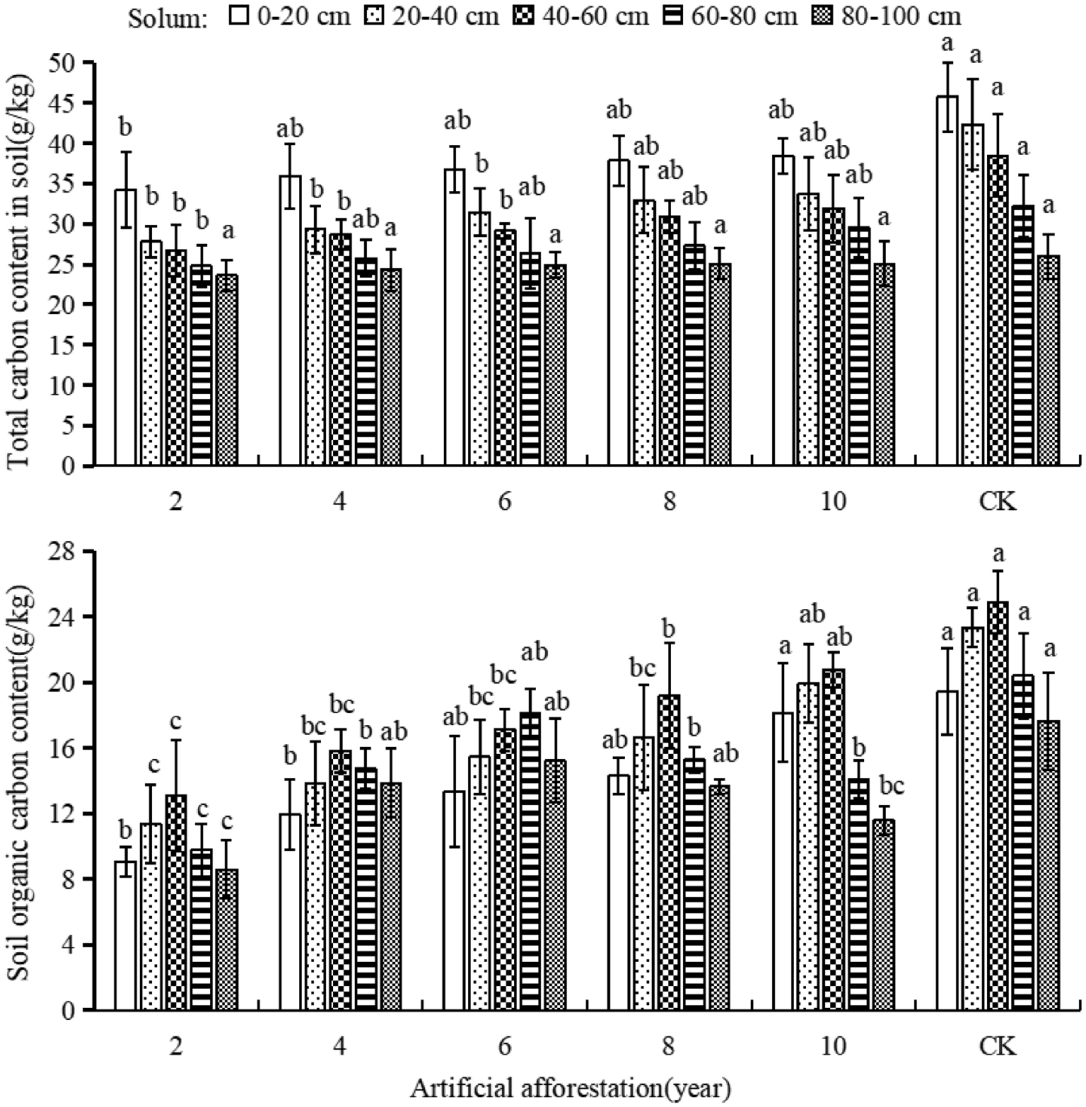
Soil total carbon and organic carbon content in different years of afforestation in Maxian Mountain of the Loess Plateau. Different lowercase letters indicate significant differences in soil total carbon and organic carbon content at the *P* < 0.05 level for the same soil layer and different years of afforestation.

In the Maxian Mountain of the Loess Plateau, the total carbon content of soil in the same soil layer gradually increased as the years of afforestation prolonged. Soil organic carbon content increased in the soil layers of 0–20, 20–40, and 40–60 cm, but decreased in the 60–80 and 80-100 cm (Fig. 4). In terms of soil total carbon content, after 2 years of afforestation, each soil layers were significantly smaller than CK. The total carbon content of afforestation for 4 and 6 years was significantly smaller in soil layers of 20–40 and 40–60 cm compared to CK (*P* < 0.05). When the afforestation period was 8 or 10 years, there was no significant difference between the soil layers and CK. In terms of soil organic carbon content, after two years of afforestation, the soil layers of 0–20, 20–40, 40–60, 60–80, and 80–100 cm were significantly smaller than CK. When the afforestation period was 4, 6, or 8 years, the soil layers of 20–40 and 40–60 cm were significantly lower compared with CK. A 10-year afforestation period resulted in significantly lower in soil layers of 60–80 and 80–100 cm compared to CK (*P* < 0.05). All the plots, the total carbon content of the soil gradually decreased with the soil depth increased, while the organic carbon content initially increased and then decreased. This indicated that with the increasing of afforestation years, the soil total carbon content gradually increased and approached CK. However, the soil organic carbon content gradually increased in the soil layers of 0–20, 20–40, and 40–60 cm, while it decreased in the 60–80 and 80–100 cm.

### The effect of afforestation years on soil nitrogen

In the Maxian Mountain of the Loess Plateau, with the increasing of afforestation years, the total nitrogen content in each soil layers increased first and then decreased, whereas the soil nitrate and ammonium nitrogen content gradually increased and approached CK (Table 2). Two years of afforestation resulted in significantly lower soil total nitrogen content compared to CK (*P* < 0.05). Afforestation periods of 2 or 4 years resulted in significantly lower soil nitrate and ammonium nitrogen levels compared to periods of 8 years, 10 years, and CK (*P* < 0.05). As the afforestation period increased by 2, 4, 6, and 8 years, the soil total nitrogen, nitrate-nitrogen, and ammonium nitrogen levels gradually increase and approach CK. The total nitrogen content decreased as the soil layer deepened over the same afforestation years. When the afforestation period was two, four, or 6 years long, the soil nitrate nitrogen content decreased as the soil layers deepen. It increased and then decreased with soil layer depth during the afforestation periods of 8, 10 years, and CK. During the 2-year, 4-year, and CK, the ammonium nitrogen content of soil decreased with soil layer depth. During the 6, 8, and 10 years of afforestation, it increased first and then decreased with the depth of the soil layer. The duration of afforestation did not affect the vertical distribution pattern of soil total nitrogen content but influenced the vertical distribution state of soil nitrate nitrogen and ammonium nitrogen.

**Table 2:** Soil total nitrogen, soil nitrate nitrogen, and soil ammonium nitrogen content of different artificial forestation years in the Maxian Mountain of the Loess Plateau.

Afforestation (year)	Solum (cm)	Soil total nitrogen (g/kg)	Soil nitrate nitrogen (mg/kg)	Soil ammonium nitrogen (mg/kg)
2	0–20	1.2260 ± 0.1422b	3.7196 ± 0.3786c	13.0775 ± 1.3275d
	20–40	1.0837 ± 0.1869b	3.2454 ± 0.9990d	12.5914 ± 1.5019b
	40–60	0.9978 ± 0.1276b	3.2238 ± 0.4561d	12.5040 ± 1.6150b
	60–80	0.8729 ± 0.2065b	2.8706 ± 0.6415d	12.1868 ± 1.0542b
	80–100	0.7646 ± 0.1318b	2.7275 ± 0.3332d	10.7403 ± 0.7880b
4	0–20	1.5247 ± 0.2153ab	4.6734 ± 0.2303c	15.7761 ± 1.0222cd
	20–40	1.4035 ± 0.1932ab	4.3889 ± 0.4644cd	15.6282 ± 1.3113b
	40–60	1.2105 ± 0.0651a	4.0753 ± 0.6592d	15.1830 ± 1.9001b
	60–80	1.0211 ± 0.1507ab	3.9572 ± 0.5735d	14.1437 ± 1.3905b
	80–100	0.8666 ± 0.1793ab	3.8572 ± 0.6019cd	12.0083 ± 1.6859b
6	0–20	1.7119 ± 0.3941a	7.4229 ± 0.7714bc	18.5617 ± 1.6863bc
	20–40	1.5587 ± 0.3033ab	7.2052 ± 0.6782c	20.5883 ± 2.1255a
	40–60	1.3897 ± 0.2166a	6.8819 ± 0.3755c	20.8321 ± 2.6838a
	60–80	1.1903 ± 0.2198ab	6.0974 ± 0.3987c	20.2509 ± 2.1074a
	80–100	0.9545 ± 0.2244ab	4.8903 ± 0.5784c	18.4561 ± 1.3692a
8	0–20	1.6133 ± 0.3201ab	9.1891 ± 1.8150ab	20.7231 ± 1.4979ab
	20–40	1.4199 ± 0.3896ab	10.1494 ± 1.9557b	21.9028 ± 1.8746a
	40–60	1.2205 ± 0.1743a	9.1508 ± 1.1144b	22.6280 ± 1.2557a
	60–80	1.0640 ± 0.2644ab	7.9966 ± 0.4902b	21.6511 ± 1.7442a
	80–100	0.9656 ± 0.1921ab	7.5385 ± 0.6027b	20.0733 ± 1.4530a
10	0–20	1.5766 ± 0.2546ab	10.3871 ± 1.7645ab	23.5887 ± 1.9150a
	20–40	1.2897 ± 0.1605ab	11.4550 ± 1.1818b	23.9538 ± 1.1349a
	40–60	1.1399 ± 0.1944ab	10.7930 ± 1.4642b	22.4818 ± 2.1377a
	60–80	0.9545 ± 0.1646ab	8.0440 ± 0.6828b	20.2102 ± 1.4739 a
	80–100	0.9092 ± 0.1569ab	7.5117 ± 1.3509b	17.4113 ± 0.9135 a
CK	0–20	1.7911 ± 0.3559a	13.4399 ± 1.2399a	23.5954 ± 1.5791a
	20–40	1.6635 ± 0.2372a	14.7083 ± 1.7654a	23.1273 ± 1.6116a
	40–60	1.5521 ± 0.3151a	13.6351 ± 0.8596a	22.5284 ± 2.0195a
	60–80	1.4404 ± 0.2051a	12.6251 ± 1.2800a	21.9541 ± 1.7464a
	80–100	1.3554 ± 0.2550a	11.9259 ± 1.2436a	20.2842 ± 1.5707a

Different lowercase letters indicate significant differences in soil total nitrogen, soil nitrate nitrogen, and soil ammonium nitrogen content at the *P* < 0.05 level for the same soil layer but different years of afforestation.

### The effect of afforestation years on soil phosphorus

**Figure 5:**
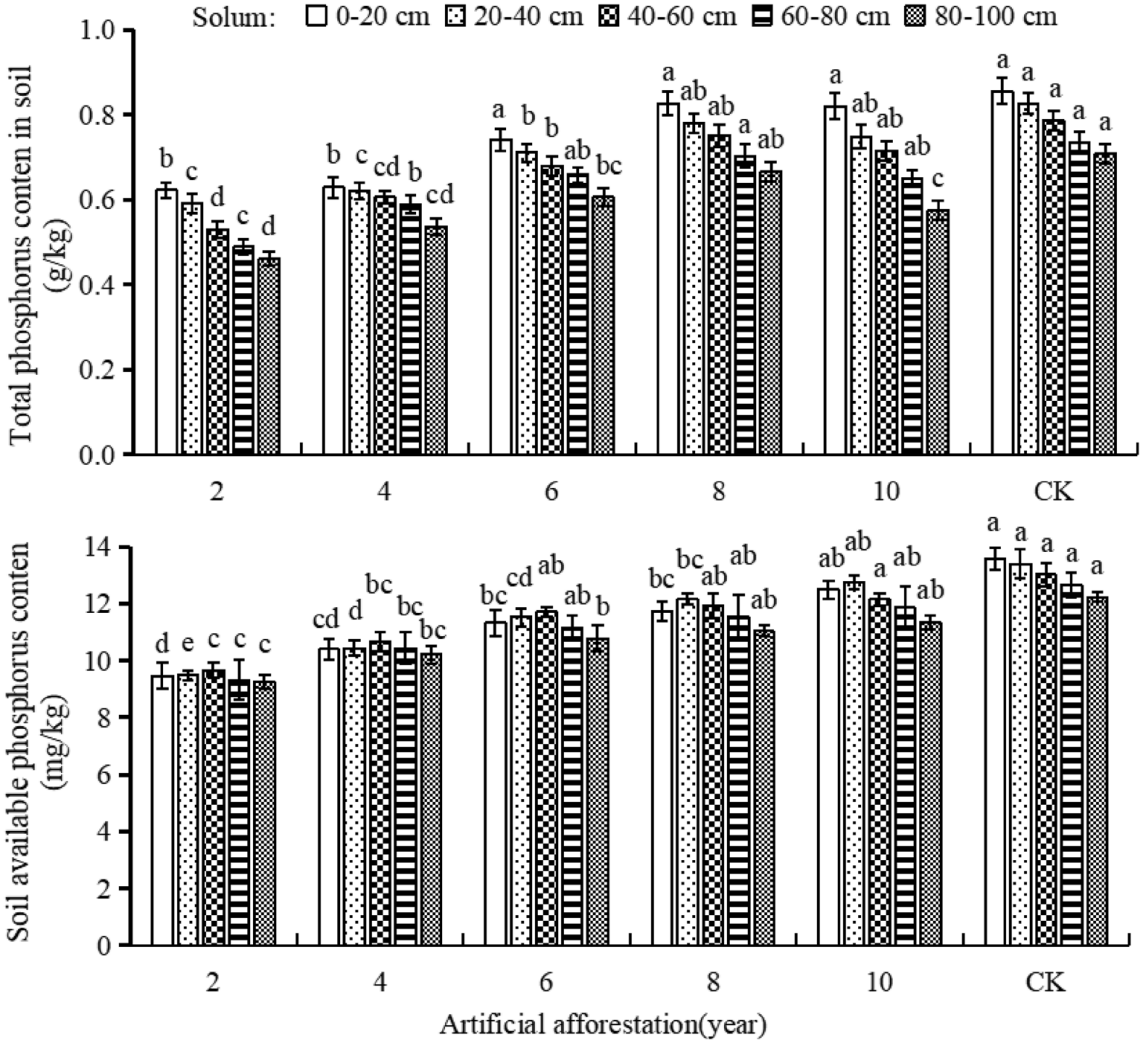
Soil total phosphorus and available phosphorus content in different years of afforestation in Maxian Mountain of the Loess Plateau. Different lowercase letters indicate significant differences in soil total phosphorus and available phosphorus content at the *P* < 0.05 level for the same soil layer and different years of afforestation.

In Maxian Mountain of the Loess Plateau, with the increasing of afforestation years, the total phosphorus content in each soil layers increased and then decreased, while the available phosphorus content increased (Fig. 5). The afforestation period was 2 years, and the total and available phosphorus content in the same soil layer was significantly lower than the afforestation periods of 6, 8, 10, and CK. Four years of afforestation resulted in significantly lower total and available phosphorus levels in the same soil layer compared to CK (*P* < 0.05). This indicates that the afforestation period increased over 2, 4, 6, and 8 years, with an increasing in total phosphorus and available phosphorus content close to CK. However, when the afforestation period was 10 years, soil total phosphorus content decreased.The afforestation period was 2, 4, 6, 8, and 10 years, total phosphorus content of CK soil decreased with the depth of soil layer. The soil available phosphorus content in all afforestation plots increased first and then decreased with the depth of soil layer. When the afforestation period was 2, 4, and 6 years, the highest content of available phosphorus occurred in the 40–60 cm soil layer. When the afforestation period was 8 or 10 years, the highest content of available phosphorus occurs in the 20–40 cm soil layer. The length of afforestation years could not change the vertical distribution pattern of soil total phosphorus content but affected the vertical distribution status of soil available phosphorus content.

### The effect of afforestation years on soil potassium

**Figure 6:**
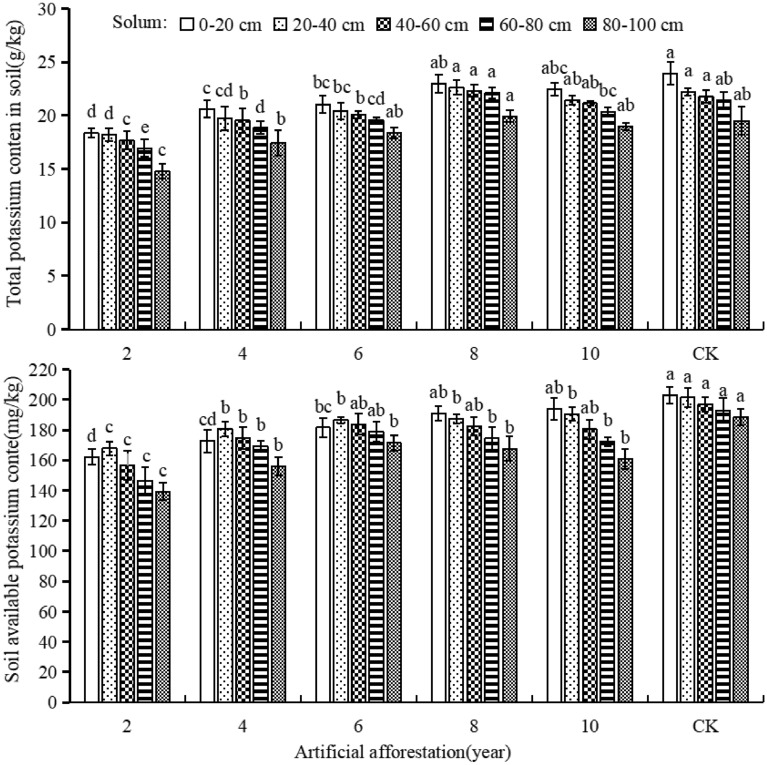
Soil total potassium and available potassium content in different years of afforestation in Maxian Mountain of the Loess Plateau. Different lowercase letters indicate significant differences in soil total potassium and available potassium content at the *P* < 0.05 level for the same soil layer and different years of afforestation.

As the increasing of afforestation years, the total potassium content in each soil layers increased first and then decreased, whereas the available potassium content increased in the soil layers of 0–20 and 20–40 cm and then decreased in the 40–60, 60–80, and 80–100 cm (Fig. 6). In the same soil layer, the total potassium content of afforestation observed on 8, 10, 6, 4, and 2 years. There was no significant difference between afforestation years of 8 years, 10 years, and CK, but all were significantly higher than 2 years of afforestation (*P* < 0.05). In the same soil layer, the available potassium content was significantly decreased after 2 years of afforestation than 6, 8, 10 years, and CK. This suggests that as the afforestation period prolongs, the total and available potassium contents in the soil rised and approached CK. However, after 10 years of afforestation, the total and available potassium content in soil layers of 40–60, 60–80, and 80–100 cm decreased. The total potassium content of the soil decreased as the soil layer deepened over time due to afforestation and CK. The afforestation period was 8, 10 years, and CK, and the soil-available potassium content decreased as the soil layer deepened. However, the afforestation period lasts 2, 4, and 6 years, and the available potassium content increases as the soil layer deepens before decreasing. The duration of afforestation did not affect the vertical distribution pattern of soil total potassium, but affected the vertical distribution of soil available potassium content in sample plots with afforestation duration of 2, 4, and 6 years.

### The influence of afforestation years on the comprehensive index of soil fertility

**Figure 7:**
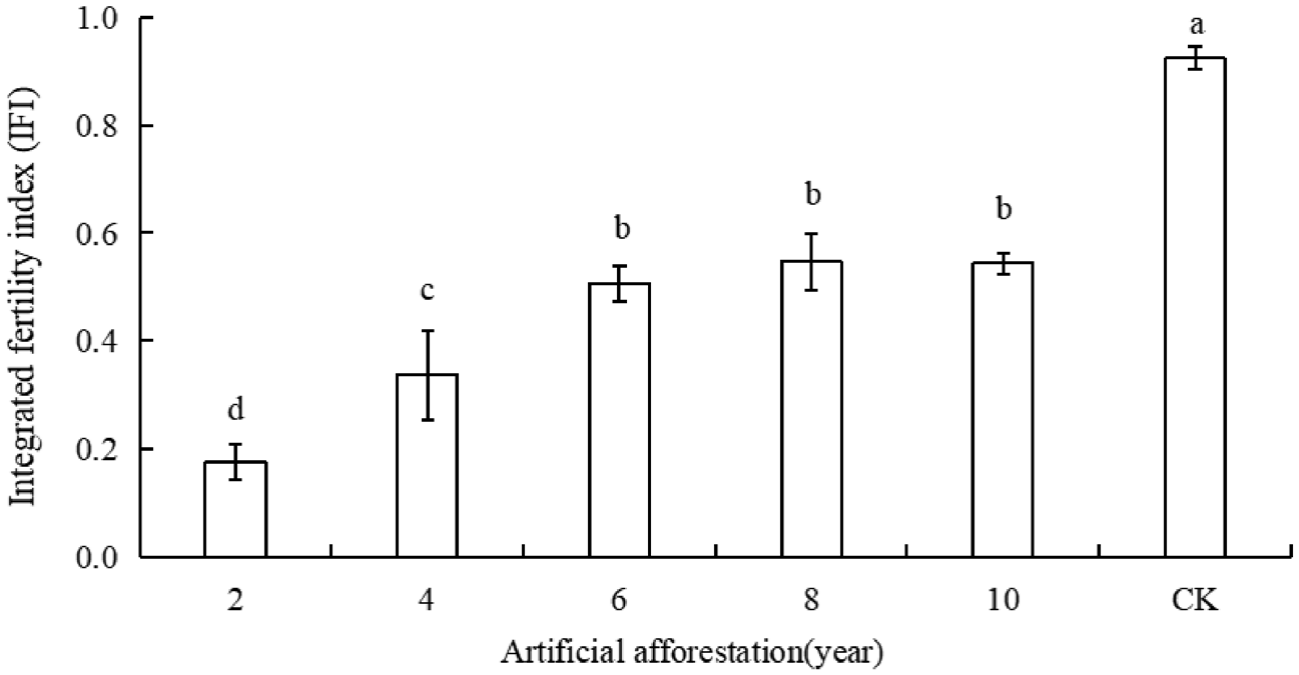
Soil fertility comprehensive index of different years of afforestation in Maxian Mountain of the Loess Plateau. Different lowercase letters indicate different years of afforestation, and the significant difference in soil fertility comprehensive index at the *P* < 0.05 level.

The integrated fertility index (IFI) in Maxian Mountain of the Loess Plateau increased first and then decreased with the prolonging of afforestation years (Fig. 7). The values of IFI in different afforestation years were ranked as follows: 8 years > 6 years > 10 years > 4 years > 2 years. The afforestation period of 2 years was significantly less than 4 years, and the afforestation period of 2 and 4 years was significantly less than the afforestation period of 6, 8, and 10 years. All afforestation plots were significantly less than CK (*P* < 0.05).

## Discussion

Sufficient soil moisture content is necessary for trees to grow. However, large-scale afforestation has lowered surface temperatures, making the local climate warmer and drier^35^. Planting water-consuming plants in severely water-scarce areas has also hampered plant growth and development^36^. Therefore, improper planting causes soil moisture loss, resulting in negative feedback between vegetation growth and soil water holding capacity, leading to soil drying and layering^37^. This study indicated that as the number of years afforestation increased over a 10-year period in Maxian Mountain of the Loess Plateau, the soil moisture content increased in the soil layer of 0–20 cm, while deceased in the 20–40, 40–60, 60–80, and 80–100 cm. Moreover, the soil moisture content deceased with the soil layer depth increased. This was because precipitation accounts for the majority of soil moisture content on the Loess Plateau. Artificial forests and under-story vegetation can effectively intercept precipitation, increasing the surface soil moisture content. However, as the number of years of afforestation increased, the tree roots develop, and the ecological water demand increased. When precipitation is insufficient to support tree growth, deep soil moisture is absorbed and consumed by the roots, resulting in continuous soil moisture deficiency and a soil drying layer. This is consistent with the findings of Wang, Wu, and others. The excessive large-scale planting of Caragana, Pinus tabulaeformis, and Robinia pseudoacacia on the Loess Plateau depletes soil moisture, promoting the soil drying layer phenomenon^38,39^. In Maxian Mountain on the Loess Plateau, the increasing duration of afforestation reduces the soil water content and limits the growth of trees.

Soil pH is an important indicator for evaluating soil quality^40^. Excessive or insufficient pH can affect soil nutrient absorption and utilisation, influencing plant growth and development^41^. Soil acidification causes the release of toxic elements, reduces the variety and quantity of microorganisms, impedes nutrient cycling, and inhibits plant growth^42^. Alkaline soil contains a high salt content, has low permeability, and nutrient elements are fixed or oxidised, making it difficult for plant roots to absorb water and nutrients, resulting in “physiological drought” and “nutrient deficiency syndrome”^43^. At the same time, soil pH is influenced by moisture content, with arid soils typically having a higher pH than moist soils^44^. This study shows that in the Maxian Mountain area of the Loess Plateau, with the same number of years of afforestation, the soil pH rises with depth of the layer. However, the pH of the 0–20, 20–40, 40–60, 60–80, and 80–100 cm soil layers rises as the number of years of afforestation increases. This is due to the decomposition of artificial forest litter, which produces organic acids that seep into the soil with rainfall, resulting in lower alkalinity on the surface soil. In addition, base ions leach from the upper layer of soil and accumulate in the lower layer, neutralizing acidic substances produced during soil organic matter decomposition and inhibiting soil acidification. Soil moisture content and pH have a synergistic effect. Soil moisture is absorbed and consumed by trees year after year, forming a soil-drying layer that causes soil pH to rise as the number of years of afforestation increases. This is consistent with research findings in the Qilian Mountain of China^45^, the Inner Mongolian Plateau^46^, the North China Plain^47^, the Beijing Yanqing Forest Region^48^, and elsewhere, which show that the soil pH of artificial forests rises as soil layers deepen and the forest ages.

Carbon, nitrogen, phosphorus, and potassium in soil are essential elements for plant growth and development^49^. The total nutrient content of soil carbon, nitrogen, phosphorus, and potassium indicates the potential extent of soil fertility. The amount of effective nutrients in soil specifically carbon, nitrogen, phosphorus, and potassium directly regulates the normal growth and reproduction of plants.

This study shows that the increase of afforestation years of 2, 4, 6, 8, and 10 years, the total carbon, total nitrogen, total phosphorus, and total potassium contents in the 0–20, 20–40, 40–60, 60–80, and 80–100 cm soil layers in the Maxian Mountain of the Loess Plateau first rise and then fall, but they are all lower than the CK sample plots. The Loess Plateau region will transform unsuitable farmland into artificial forests, which were originally a carbon source, into a forest ecosystem with a carbon sink function. Photosynthesis absorbs and fixes CO_2_ in the atmosphere, while afforestation increases the soils total carbon content of the soil^50^. Meanwhile, with the increasing number of years of afforestation, nitrogen, phosphorus, and potassium elements are returned to the soil through atmospheric deposition, litter decomposition, microbial action, etc.^51^, increasing the content of total nitrogen, total phosphorus, and total potassium. The Maxian Mountain of the Loess Plateau has the highest total carbon, phosphorus, and potassium content after 8 years of afforestation, while the total nitrogen content is highest after 6 years. The main reason for this age difference is that the soil has undergone denitration. Wang Mengjuan et al. discovered that in artificial forests with a 5-year age, denitrification bacteria in the soil are significantly stronger than in the early stages of afforestation. When the soil nitrogen content is high, denitrification bacteria convert nitrate to nitrogen gas and release it into the atmosphere, maintaining soil nitrogen balance^52^. The total carbon, total nitrogen, total phosphorus, and total potassium contents in the 0–20, 20–40, 40–60, 60–80, and 80–100 cm soil layers are lower after 10 years of afforestation than after 8 years. The phenomenon of no increase and no decrease is caused by the input of nutrients being less than the output, indicating a decrease in potential soil fertility.

Soil nutrients are available in the form of ions, which are either free in soil solution or adsorbed on the surface of the soil aggregates. They are nutrient elements that can be directly absorbed and utilized by plant roots, and their content directly reflects the strength of soil fertility. This study indicates that effective nutrients such as soil organic carbon, nitrate nitrogen, ammonium nitrogen, available potassium, and available phosphorus increase over the afforestation period of 2, 4, 6, 8, and 10 years. They continuously increase in shallow soils (0–20, 20–40, and 40–60 cm), but first increase and then decrease in deep soils (60–80 and 80–100 cm). This is because the physicochemical properties of soil in artificial forests are sensitive to changes in afforestation years^50^, especially with the increase of afforestation years, which can stimulate the release of plant root exudates^53^, enhance soil microbial activity^54^, and promote soil animal activity thereby accelerating the decomposition of soil litter and increasing soil organic carbon, available nitrogen, and available phosphorus. The content of effective nutrients such as available potassium enhances soil fertility. This method of improving soil fertility often occurs in the soil’s surface layer, because the soil permeability decreases with depth, it is unsuitable for the survival and reproduction of animals and microorganisms. Only tree root exudates improve soil nutrient effectiveness, but they continuously absorb and utilize effective nutrients, resulting in longer afforestation years and lower nutrient availability in deep soil. When the afforestation period is 10 years, the effective nutrient content in soil layers 60–80 cm and 80–100 cm is lower than in the sample plot with an afforestation period of 8 years. This finding confirms that when afforestation lasts 10 years in the Loess Plateau, the available nutrients in the deep soil shift from increasing to consuming. The vertical distribution pattern of total carbon, nitrogen, phosphorus, and total potassium in soil samples with afforestation years of 2, 4, 6, 8, and 10 years decreased with increasing soil depths of 0–20, 20–40, 40–60, 60–80, and 80–100 cm, which is consistent with the CK. This is due to the surface aggregation effect of trees on soil nutrients^55^, which results in increased nutrient content in the surface soil. Simultaneously, the growth and decline of the total nutrient content of soil carbon, nitrogen, phosphorus, and potassium in artificial forests are determined by the soil parent material, and the duration of afforestation is significantly shorter than that of soil weathering. Therefore, the duration of afforestation has no effect on the vertical distribution pattern of the total nutrient content of soil carbon, nitrogen, phosphorus, and potassium. The vertical distribution pattern of soil organic carbon content in plots with afforestation years of 2, 4, 6, 8, and 10 years shows an initial increase followed by a decrease, while the vertical distribution pattern of soil available nutrients such as nitrate nitrogen, ammonium nitrogen, available phosphorus, and available potassium varies. Because artificial forests helps to fix organic carbon through photosynthesis, maintain dynamic balance of vertical distribution patterns, and are consistent with CK. The duration of afforestation determines the quantity of litter and the distribution of tree roots, regulates the content of available nutrients such as nitrogen, phosphorus, and potassium in the soil, disrupts the vertical distribution pattern of available nutrients in the soil, and leads to differentiation from CK.

The integrated fertility index in the Loess Plateau increased first and then decreased as the number of years of afforestation increasing, but is significantly lower than CK. When the afforestation period is 2, 4, 6, or 8 years, the soil moisture and nutrient content increase, promoting the improvement of soil fertility. However, when the afforestation period is 10 years, the total amount of litter in the artificial forest is small and fluctuates greatly, resulting in an imbalance between the return amount and absorption amount, leading to soil degradation^56^. The soil on the Loess Plateau is barren, and afforestation is subjective, singular, and limited when compared to natural restoration. It necessitates the absorption and utilization of additional soil moisture and nutrients, which is part of a rapid ecological restoration strategy. The soil fertility has been depleted over time and cannot be restored at its natural location, which is confirmed by Berthrong^57^.

When the afforestation period in the Maxian Mountain of the Loess Plateau was 10 years, the seedlings of *Picea crassifolia* led to the loss of restored soil fertility. This is due to the fact that *P. crassifolia* is in the young forest stage from 0 to 40 years old^58^, and the growth is vigorous and requires more nutrient requirements. In addition, the lack of soil water and fertilizer on the Loess Plateau restricted the growth of trees, resulting in canopy density and difficulty in becoming a forest. However, in a longer time range, for example, when the 41–100-year-old *P. crassifolia* is in the middle-aged forest, near-mature forest, and mature forest, with the decline of the tree’s nutrient demand, whether it will provide positive feedback to the soil and increase fertility needs to be further studied.

## Conclusion

In the current study, soil fertility in the Maxian Mountain of the Loess Plateau recovered slowly at 2 or 4 years and faster at 6 and 8 years, but began to decrease at 10 years, indicating that the inflection point of soil fertility from positive to negative was 10 years. Therefore, after 10 years, the seedlings planted in the Maxian Mountain of the Loess Plateau evolved into “pumping machines” that continuously consumed soil water and fertilizer, resulting in a vicious circle of seedlings that were difficult to grow into forests and soil fertility declined. In view of this, it is necessary to follow the principle of “suitable trees in the right place” and carry out ecological restoration in poor soil fertility areas, which should be based on the premise of protecting and improving soil fertility.

## Data Availability

Data are available from the corresponding author on request.
